# Does Mortality Risk of Cigarette Smoking Depend on Serum Concentrations of Persistent Organic Pollutants? Prospective Investigation of the Vasculature in Uppsala Seniors (PIVUS) Study

**DOI:** 10.1371/journal.pone.0095937

**Published:** 2014-05-14

**Authors:** Duk-Hee Lee, Lars Lind, David R. Jacobs, Samira Salihovic, Bert van Bavel, P. Monica Lind

**Affiliations:** 1 Department of Preventive Medicine, School of Medicine, Kyungpook National University, Daegu, Korea; 2 BK21 Plus KNU Biomedical Convergence Program, Department of Biomedical Science, Kyungpook National University, Daegu, Korea; 3 Department of Medical Sciences, Cardiovascular Epidemiology, Uppsala University Hospital, Uppsala, Sweden; 4 Division of Epidemiology and Community Health, School of Public Health, University of Minnesota, Minneapolis, Minnesota, United States of America; 5 Department of Nutrition, University of Oslo, Oslo, Norway; 6 MTM Research Center, School of Science and Technology, Örebro University, Örebro, Sweden; 7 Department of Medical Sciences, Occupational and Environmental Medicine, Uppsala University, Uppsala, Sweden; Kyushu University Faculty of Medical Science, Japan

## Abstract

Cigarette smoking is an important cause of preventable death globally, but associations between smoking and mortality vary substantially across country and calendar time. Although methodological biases have been discussed, it is biologically plausible that persistent organic pollutants (POPs) like polychlorinated biphenyls (PCBs) and organochlorine (OC) pesticides can affect this association. This study was performed to evaluate if associations of cigarette smoking with mortality were modified by serum concentrations of PCBs and OC pesticides. We evaluated cigarette smoking in 111 total deaths among 986 men and women aged 70 years in the Prospective Investigation of the Vasculature in Uppsala Seniors (PIVUS) with mean follow-up for 7.7 years. The association between cigarette smoking and total mortality depended on serum concentration of PCBs and OC pesticides (P value for interaction = 0.02). Among participants in the highest tertile of the serum POPs summary score, former and current smokers had 3.7 (95% CI, 1.5–9.3) and 6.4 (95% CI, 2.3–17.7) times higher mortality hazard, respectively, than never smokers. In contrast, the association between cigarette smoking and total mortality among participants in the lowest tertile of the serum POPs summary score was much weaker and statistically non-significant. The strong smoking-mortality association observed among elderly people with high POPs was mainly driven by low risk of mortality among never smokers with high POPs. As smoking is increasing in many low-income and middle-income countries and POPs contamination is a continuing problem in these areas, the interactions between these two important health-related issues should be considered in future research.

## Introduction

Cigarette smoking, as a major risk factor for cancer, cardiovascular diseases, and pulmonary diseases, is an important cause of preventable death globally [1]. However, the magnitude of associations between cigarette smoking and mortality varies across countries and calendar time [2], [3], [4], [5]. To date, this variation has been attributed mainly to methodological biases such as misclassification of smoking status, cohort effects, or random variation [2], [3], [4], [5].

In our previous study [6], we formulated a novel hypothesis that persistent organic pollutants (POPs) modify the risk of cigarette smoking on death. POPs are lipophilic chemicals that accumulate in adipose tissue and are associated with the risk of various chronic diseases [7], [8], [9]. The biological plausibility for our hypothesis was that experimental studies in mice reported that pretreatment with some POPs increased toxicity of important chemicals contained in cigarette smoke, like benzopyrene, dimethylnitrosamine, and N-nitrosodiethlyamine [10], [11].

Supporting our prior hypothesis, we observed different associations between cigarette smoking and total mortality depending on serum concentrations of POPs among the elderly in the U.S. [6]. In that study, one surprising finding was that cigarette smoking did not increase the risk of mortality in the lowest category of POPs. Despite the possibility that smoking-related diseases risks are lower among the elderly than in younger persons due to selective survival [12], finding any subgroup with no association between cigarette smoking and mortality was unexpected.

Our earlier study is the only one published on this topic and the number of current smokers was too small [6]. Thus, we studied here whether the finding was replicable in another dataset. We evaluated if there are similar interactions of serum concentrations of POPs with cigarette smoking on the risk of mortality among men and women aged 70 years, living in the community of Uppsala, Sweden (Prospective Investigation of the Vasculature in Uppsala Seniors (PIVUS) study). Because our previous study found that, among various POPs, polychlorinated biphenyls (PCBs) or organochlorine (OC) pesticides showed clear interactions with cigarette smoking and mortality [6], we used POPs burden calculated based on serum concentrations of both OC pesticides and PCBs in this study.

## Materials and Methods

Study subjects at baseline were 1,016 men and women, residents of Uppsala, Sweden and age 70 years at time of examination between April 2001 and June 2004. Among participants at baseline, 986 subjects had valid measurement of PCBs and OC pesticides at baseline. The study was approved by the Ethics Committee of the University of Uppsala. The participants gave written informed consent.

All subjects were investigated in the morning after an overnight fast, with no medication or smoking allowed after midnight. The participants were asked about their health behaviors, medical history, and regular medication. Body mass index (BMI) was derived from measured height and weight (kg/m^2^). Serum cholesterol and triglyceride concentrations were determined in an enzymatic assay (Abbott, Abbott Park, IL, USA).

POPs were measured in stored plasma samples collected at baseline. Analyses of POPs were performed using a Micromass Autospec Ultima (Waters, Mildford, MA, USA) high resolution chromatography coupled to high resolution mass spectrometry (HRGC/HRMS) system based on the method by Sandau et al [13] with some modifications. All details of POPs analyses were provided elsewhere [14]. Among 16 PCB congeners and 5 OC pesticides, 2 OC pesticides (trans-chlordane and cis-chlordane) with detection rate <10% were not included in the final analyses. An established summation formula based on serum cholesterol and serum triglyceride concentrations was used to calculate the total amount of lipid in each plasma sample [15]. Thereafter the wet-weight concentrations of the POPs were divided by this lipid estimate to obtain lipid-normalized concentrations. As models based on wet-weight concentrations adjusted for serum cholesterol and triglyceride as covariates showed similar results, we presented the results based on lipid-normalized concentrations for the consistency of analytic strategy with the previous study [6].

The fact of death was ascertained through linkage to the Swedish Register of Death Causes at the National Board of Health and Welfare. Causes of death data were not available. Follow-up time for each person was calculated as the difference between the first examination date and the last known date alive or censored. Persons who survived the entire follow-up period were censored on Jan 1, 2012. Median follow-up time was 7.7 years (range 0.3–9.8 years) and we documented 111deaths.

For the analysis, smoking status was expressed as never, former and current. First, we calculated the summary measure of 16 PCB congeners and 5 OC pesticides by summing the rank orders of the individual POPs for subjects with detectable values of each POP, assigning rank 0 to not detectable values. Three summary measures were made based on both PCBs and OC pesticides, PCBs only, and OC pesticides only.

Next, we checked if the associations between cigarette smoking and mortality differed by tertiles of the summary measures of POPs in predicting total mortality using Cox proportional hazard models. P values for interaction were calculated based on three categories of each of POPs and cigarette smoking. Adjusting covariates were gender, physical activity (none, moderate, and vigorous), BMI (kg/m^2^), and alcohol consumption (g/day). We further considered medication for diabetes or hypertension and history of myocardial infarction or stroke as possible confounders.

In addition to stratified analyses, adjusted HRs with the common reference group of never smokers within the 1^st^ tertile of POPs were presented. Also, the same analyses were applied to the U.S. elderly within our previous study [6] because only results stratified analyses by POPs levels were previously reported in that study. Methodologic details have been presented [6]. All statistical analyses were performed with PC-SAS version 9.1.

## Results

Baseline characteristics and history of chronic diseases according to cigarette smoking status were shown in Table 1. Compared to never or current smokers, former smokers tended to be men, more obese, alcohol drinkers, and more under diabetes medication. Current smokers were less obese and more physically inactive than never or former smokers. There were no statistical significant differences across smoking categories for prevalence of hypertension medication or history of myocardial infarction or stroke.

**Table 1:** Baseline characteristics according to the status of cigarette smoking among 986 elderly aged 70, Prospective Investigation of the Vasculature in Uppsala Seniors (PIVUS) study.

	Status of cigarette smoking			
Characteristics	Never smokers (N = 471)	Former smokers (N = 410)	Current smokers (N = 105)	p value
Men (%)	44.4	57.6	47.6	0.01
BMI (kg/m^2^, %)				<0.01
<25	34.4	26.6	51.4	
25-<30	44.8	48.1	34.3	
≥30	20.8	25.4	14.3	
Exercise (%)				0.01
No	8.5	11.7	18.1	
Mild	64.1	63.4	66.7	
Moderate or vigorous	27.4	24.9	15.2	
Alcohol consumption (g/day, %)				<0.01
0	17.2	11.5	14.3	
1–14	75.2	74.8	80.0	
≥15	7.6	13.7	5.7	
Diabetes medication (%)	4.7	8.8	4.8	0.03
Hypertension medication (%)	29.5	33.4	27.6	0.34
History of myocardial infarction (%)	5.7	8.3	10.7	0.13
History of stroke (%)	3.2	3.9	4.8	0.69

Table 2 indicates mean concentrations of individual POPs depending on cigarette smoking status. Among 16 PCBs, PCB074, PCB105, and PCB118 were statistically significant or marginally significantly lower in current vs. never smokers. These three PCBs have weak dioxin activity, however, other PCBs with dioxin activity like PCB126, PCB156, and PCB169 did not show any trend. On the contrary, among 3 OC pesticides, *p,p′*-DDE had higher levels among current than never smokers.

**Table 2:** Adjusted* serum concentrations (geometric means, ng/g lipid) of individual polychlorinated biphenyls (PCBs) or organochlorine (OC) pesticides according to the status of cigarette smoking, Prospective Investigation of the Vasculature in Uppsala Seniors (PIVUS) study.

	Status of cigarette smoking			
Analytes	Never smokers (N = 471)	Former smokers (N = 410)	Current smokers (N = 105)	p for trend
**Polychlorinated biphenyls (PCBs)**				
PCB074†	14.1	13.6	12.9	0.09
PCB099	13.4	13.7	14.3	0.38
PCB105†	5.0	5.0	4.1	0.02
PCB118†	31.0	30.7	25.0	<0.01
PCB126†	5.9	5.9	6.5	0.44
PCB138	123.7	124.5	135.4	0.15
PCB153	213.7	216.5	228.3	0.19
PCB156†	23.7	23.1	24.7	0.78
PCB157	4.4	4.3	4.5	0.70
PCB169†	25.8	25.9	26.7	0.52
PCB170	75.5	75.3	80.1	0.30
PCB180	177.6	177.3	187.1	0.37
PCB189	3.2	3.2	2.9	0.34
PCB194	16.0	15.2	17.2	0.88
PCB206	4.2	3.9	4.1	0.23
PCB209	4.0	3.8	4.0	0.36
**Organochlorines pesticides (OCPs)**				
p,p′-DDE	270.1	296.4	322.4	0.04
Trans-nonachlor	20.6	21.9	21.8	0.14
Hexachlorobenzene	40.1	39.1	39.5	0.46

*Adjusted for gender, BMI, exercise, and alcohol consumption.

† POPs with dioxin activity.

When POPs were not considered in analyses, adjusted hazard ratios (HRs) for all-cause mortality were 1.3 for former smokers and 2.1 (95% CI: 1.2–3.7) for current smokers, compared with never smokers. However, the associations were substantially different depending on summary measures of POPs (Table 3). Compared with 70 year old people within the 1^st^ or 2^nd^ tertiles of summary measures of PCBs and OC pesticides, those within the 3^rd^ tertile showed strong associations between cigarette smoking and total mortality. Adjusted HRs were 3.7 (1.5–9.3) for former smokers and 6.4 (2.3–17.7) for current smokers. Further adjustment for medication for diabetes or hypertension and history of myocardial infarction or stroke did not change the result. Summary measures calculated from PCBs or OC pesticides separately showed similar associations (Table S1).

**Table 3:** Adjusted hazard ratios (HRs)* and 95% confidence intervals (CIs) for all-cause mortality rate by summary measures† of polychlorinated biphenyls (PCBs) or organochlorine (OC) pesticides, Prospective Investigation of the Vasculature in Uppsala Seniors (PIVUS) study.

		Status of cigarette smoking			p for trend	p for interaction
		Never smokers	Former smokers	Current smokers		
**All subjects**						
	Cases/No	40/471	50/410	21/105		
	Adjusted HR(95%CI)	Referent	1.3(0.9–2.0)	2.1(1.2–3.7)	<0.01	
**Summary measure of 16 PCBs and 3 OC pesticides**						
1^st^ tertile	Cases/No	16/160	19/138	5/30		
	Adjusted HR(95%CI)	Referent	1.2 (0.6–2.4)	1.4 (0.5–4.0)	0.46	0.02
2^nd^ tertile	Cases/No	18/161	10/134	5/34		
	Adjusted HR(95%CI)	Referent	0.7 (0.3–1.6)	1.2 (0.5–3.4)	0.97	
3^rd^ tertile	Cases/No	6/150	21/138	11/41		
	Adjusted HR(95%CI)	Referent	3.7 (1.5–9.3)	6.4 (2.3–17.7)	<0.01	

*Hazard Ratios (HRs) adjusted for gender, BMI, exercise, and alcohol consumption.

† Values of compounds belonging to in each summary measure were individually ranked; the rank orders of the individual POPs were summed to calculate summary measures and the summaries were divided into tertiles.

**Figure 1:**
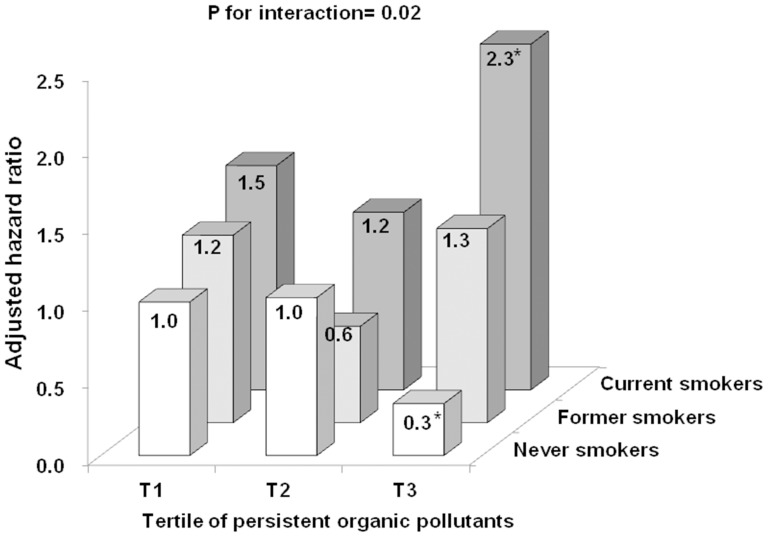
Interactions of cigarette smoking with summary measure of polychlorinated biphenyls (PCBs) and organochlorines (OC) pesticides on mortality. Hazard ratios (HRs) were estimated using a common reference group (never smokers & T1), adjusted for gender, BMI, exercise, and alcohol consumption. T1, first tertile; T2, second tertile; T3, third tertile, Prospective Investigation of the Vasculature in Uppsala Seniors (PIVUS) study.

Figure 1 shows adjusted HRs when never smokers within the 1^st^ tertile of PCBs and OC pesticides were used as the common reference group. Never smokers within the 3^rd^ tertile of PCBs and OC pesticides showed a statistically significantly lower risk of mortality with adjusted HR of 0.3 (0.1–0.9) while current smokers with the same levels of POPs concentrations had a statistically significantly higher risk of mortality with adjusted HR of 2.3 (1.0–5.0). When we applied the same analyses to the U.S. elderly who were included in our previous study, patterns were similar to those in the current study, although no individual HR reached statistical significance (Figure S1). In that study, the summary measures for PCBs and for OC pesticides were separate because PCB vs OC pesticide measurements were performed in different participants.

To better understand possible confounding, Table 4 compared baseline characteristics of 4 subgroups (never smokers & low POPs, current smokers & high POPs, never smokers & low POPs, and current smokers & high POPs), in particular focusing on never smokers with high POPs who had the lowest mortality. Compared to other subgroups, 70 year old participants within the never smokers & high POPs group were the most physically active and had the lowest prevalence of history of myocardial infarction.

**Table 4:** Comparison of baseline characteristics among 4 subgroups, Prospective Investigation of the Vasculature in Uppsala Seniors (PIVUS) study.

	Low POPs		High POPs		
	Never smokers (N = 160)	Current smokers (N = 30)	Never smokers (N = 150)	Current smokers (N = 41)	p value
Dead (%)	10.0	16.7	4.0	26.8	<0.01
Men (%)	31.9	40.0	56.0	53.7	<0.01
BMI (kg/m^2^, %)					<0.01
<25	26.3	46.7	36.0	48.8	
25-<30	40.6	36.7	51.3	46.3	
≥30	33.1	16.7	12.7	4.9	
Moderate or vigorous exercise (%)					<0.01
No	10.0	10.0	10.7	26.8	
Mild	63.8	80.0	58.7	61.0	
Moderate or vigorous	26.3	10.0	30.7	12.0	
Alcohol consumption (g/day, %)					0.03
0	20.6	20.0	14.0	7.3	
1–14	76.3	76.7	74.0	85.4	
≥15	3.1	3.3	12.0	7.3	
Diabetes medication (%)	3.8	0%	7.3	9.8	0.17
Hypertension medication (%)	31.3	30.0	31.3	22.0	0.68
History of myocardial infarction (%)	6.9	13.8	4.0	15.0	0.05
History of stroke (%)	1.9	6.7	5.3	4.9	0.35

## Discussion

Generally, the present study replicated our report in a U.S. elderly population [6]. The association between cigarette smoking and total mortality depended on serum concentration of summary scores reflecting the background mixture of PCBs and/or OC pesticides. When POPs were not considered in the analyses, the risk of mortality among current-smokers was about two times higher than never smokers. However, among elderly with relatively high POPs, former or current smokers had about 4 to 7 times higher mortality than never smokers while the association between cigarette smoking and total mortality much weaker and statistically non-significant among elderly with relatively low serum concentrations of POPs. In addition to the main results, a subsidiary finding of lower concentrations of dioxin-like PCBs among smokers than never smokers was also similar to finding in the previous study [6].

These findings suggested that different concentrations of POPs among populations may partly explain variability in smoking-related total mortality association across previous epidemiological studies [2], [3], [4], [5]. More importantly, the similar weak association between cigarette smoking and mortality among the elderly people in the lowest category of POPs from these two studies could indicate that the presence of certain levels POPs is a necessary factor for cigarette smoking to increase the risk of death in the elderly.

At first, we had expected the interaction between these two factors to be driven by the high risk of death among current smokers with high POPs. However, these two studies showed that the strong association between cigarette smoking and mortality among the elderly with high POPs had a strong component of low mortality in never smokers as well as of high mortality in former or current smokers.

The very low mortality among the 150 never smokers in PIVUS with high POPs is a provocative finding. A similar pattern of reduced mortality was apparent in the National Health and Nutrition Examination Survey (NHANES) (Figure S1), suggesting that this finding is not simply bias or chance. An ecologic finding in the cohort study of male British doctors may be viewed as concordant. In that study, the excess mortality associated with smoking was greater during the second half of follow-up (1971∼1991) than the first half (1951∼1971) [2], largely because the mortality rate among non-smokers had decreased substantially over time while the mortality rate among smokers had remained about constant [2]. Historically, PCBs and OC pesticides were widely used after World War II and periods with the highest body burden of POPs were 1960s and 1970s [16]. Therefore, any effect due to high POPs may be more strongly reflected in the latter part of cohort study.

It is difficult to explain very low mortality among never smokers with high POPs. One possible explanation is survival bias. However, for survival bias to explain this finding, never smokers with high POPs must have had a higher death rate before reaching age 70 than other subgroups like active smokers with high POP, leaving healthier survivors to participate in the PIVUS and NHANES studies. This would be an odd pattern. Also, compared to other subgroups, the elderly never smokers with high POPs were the most physically active and had the lowest prevalence of history of myocardial infarction, both of which might predispose to reduced mortality rate. Therefore, this type of survival bias would seem to be unlikely even though we cannot totally exclude it.

In addition, low mortality in relation to high POPs is biologically plausible under some conditions, a case in point relating to associations between POPs and telomeres. Telomere length has been proposed as a marker of mitotic cell age and as a general index of human aging [17]. In our previous cross-sectional study among apparently healthy Koreans [18], telomere length was increasing across serum concentrations of POPs within the lower range of POPs. In that study, the interpretation was that low dose POPs may act as a tumor promoter in carcinogenesis based on experimental findings on arsenic. For example, low concentrations of arsenic relevant to current human exposure elongated telomeres in vitro and increased *myc* and *ras* oncogenes while high concentrations of arsenic decreased telomere length [19]. As *myc* oncogene can activate telomerase [20], the authors interpreted these experimental findings on arsenic as a role of tumor promoter of low dose arsenic in human.

However, an opposite interpretation of how POPs relate to telomere length is also possible. It is well-known that shorter telomere length is associated with higher risk of early death [21]. There is a higher mortality rate, especially from heart disease and infectious disease, among elderly people who have shorter telomeres in blood DNA [22]. Therefore, in PIVUS and NHANES persons with relatively high serum POPs concentrations within background exposure levels may have had a longer survival than persons with lower serum POPs concentrations. As cigarette smoking is reported to decrease telomere length [23], longer survival with higher POPs could be observed only in never smokers, as we observed in these two studies.

Although molecular mechanisms for low dose POPs to increase telomere length have not been studied, one speculation is that certain levels of POPs might excite production of cytoprotective and restorative proteins including growth factors, phase II and antioxidant enzymes, and protein chaperones, known as hormetic effects [24]. Increased telomerase activity or slow-down of age-dependent telomere shortening has been regarded as one marker of activation of cytoprotective and restorative proteins [25]. Some previous human and experimental studies support possible beneficial effects of certain levels of POPs. For example, decreased risk of soft tissue sarcoma was reported with increased concentrations of dioxins or PCBs with dioxin activity in the general population[26]. Also, in some animal studies of low doses of TCDD or DDT, there was a tendency towards fewer tumors or altered hepatic foci than in controls, indicating an anti-carcinogenic process [27], [28]. Furthermore, decreased lipid peroxidation level in rats treated with low dose DDT was reported, compared to control rats [29].

Studies of the levels of POPs in the global environment show that emission sources of a number of POPs in the last 20 years have shifted from industrialized countries of the Northern Hemisphere to less developing countries in tropical and sub-tropical regions [30]. This is due to a late production ban on OC pesticides: some OC pesticides are still being used in agriculture and for the control of diseases, such as malaria [31]. Also, there is another active exposure source of PCBs through e-waste recycling in these countries [32]. Although the burden of cigarette smoking use is currently greatest in high-income countries, rates of smoking are increasing in many low-income and middle-income countries. Therefore, the interaction between these two important health-related issues, POPs and cigarette smoking, should be further studied from a variety of viewpoints including molecular mechanisms.

The strengths of our study include the homogenous age and community-based sampling of study subjects. There were some limitations to our study. Due to a limited sample size, we could not consider more detailed information on cigarette smoking like total pack-years or duration of smoking cessation in analyses. In addition, analyses focusing on cause-specific mortality were not possible as cause of death information was not available. Finally, even though the consistency of findings across two studies lends credence to our findings and seems to reduce the likelihood that our finding is explainable by chance or bias, our findings still require replication by other cohort studies.

This study has confirmed a strong association between cigarette smoking and mortality among elderly people with relatively high POPs, but a much weaker and not statistically significant smoking-mortality association among those with relatively low POPs. However, the observation that the lowest mortality was seen among never smokers with high POPs is provocative and requires further studies on the role of POPs in longevity. In addition more prospective studies in human, in-vitro and in-vivo experimental studies would help to elucidate potential molecular mechanisms.

## Supporting Information

Figure S1 Interactions of cigarette smoking with summary measure of polychlorinated biphenyls (PCB) or organochlorine (OC) pesticides predicting mortality in the U.S. general population (reanalyses of published results using the same National Health and Nutrition Examination Survey (NHANES) datasets (Lee, 2013). Number of study subjects and deaths were 610 and 142 for PCB analyses and 702 and 157 for OC pesticide analyses. Hazard ratios (HRs) were estimated using a common reference group (never smokers & T1), adjusted for gender, BMI, exercise, and alcohol consumption. T1, first tertile; T2, second tertile; T3, third tertile. Although none of the HRs was statistically significantly different from the reference group, as previously reported, interaction p-values were 0.008 for PCBs and 0.024 for OC pesticides.
(TIF)

Table S1
