# Supplementary figures and images for: Does Mortality Risk of Cigarette Smoking Depend on Serum Concentrations of Persistent Organic Pollutants? Prospective Investigation of the Vasculature in Uppsala Seniors (PIVUS) Study
Source: PLoS One. 2014 May 14;9(5):e95937. doi: 10.1371/journal.pone.0095937 (PMC4020745; doi:10.1371/journal.pone.0095937)

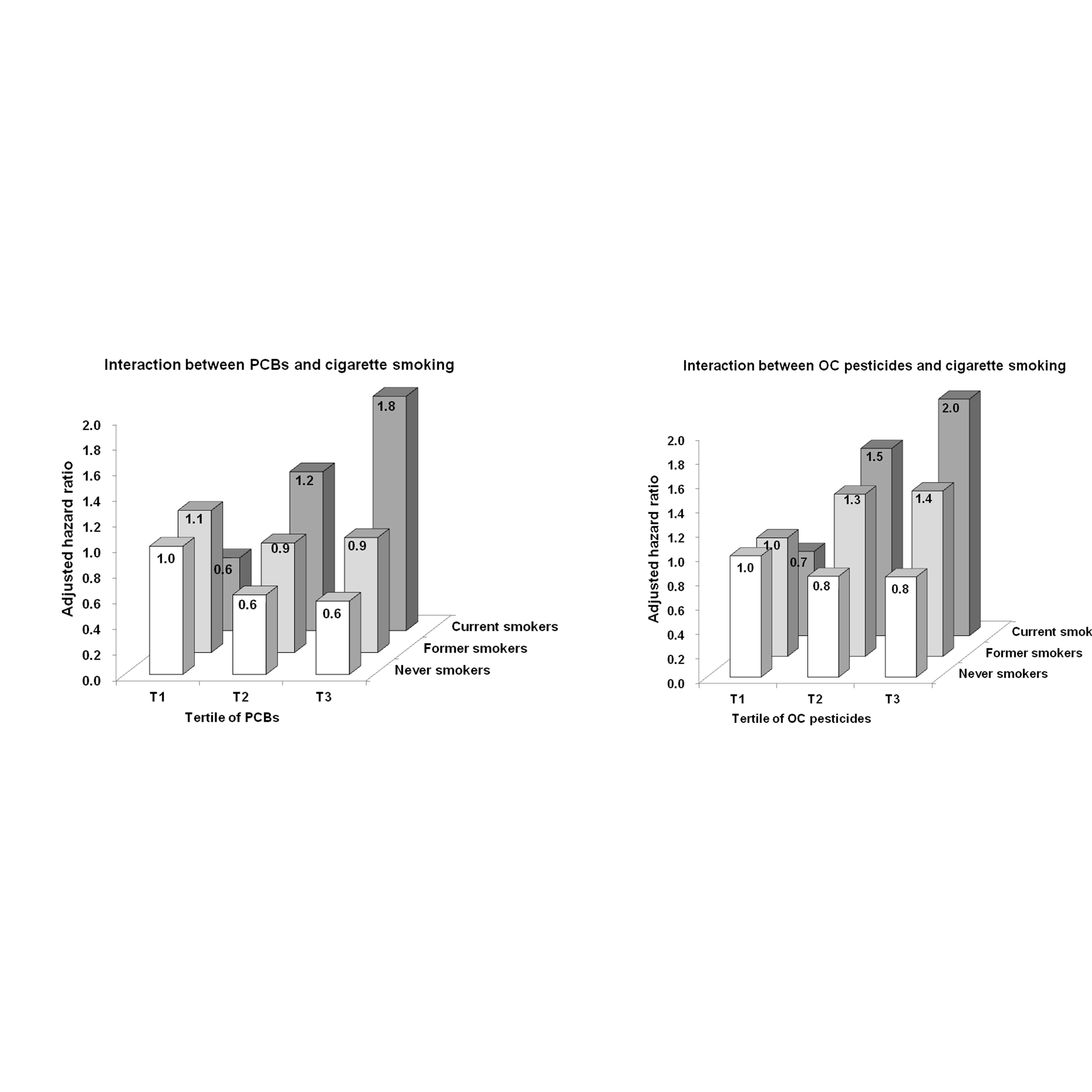

Supplement: Figure S1 — Interactions of cigarette smoking with summary measure of polychlorinated biphenyls (PCB) or organochlorine (OC) pesticides predicting mortality in the U.S. general population (reanalyses of published results using the same National Health and Nutrition Examination Survey (NHANES) datasets (Lee, 2013). Number of study subjects and deaths were 610 and 142 for PCB analyses and 702 and 157 for OC pesticide analyses. Hazard ratios (HRs) were estimated using a common reference group (never smokers & T1), adjusted for gender, BMI, exercise, and alcohol consumption. T1, first tertile; T2, second tertile; T3, third tertile. Although none of the HRs was statistically significantly different from the reference group, as previously reported, interaction p-values were 0.008 for PCBs and 0.024 for OC pesticides. (TIF) [file pone.0095937.s001.tif]
