# Supplementary material for: Does Mortality Risk of Cigarette Smoking Depend on Serum Concentrations of Persistent Organic Pollutants? Prospective Investigation of the Vasculature in Uppsala Seniors (PIVUS) Study
Source: PLoS One. 2014 May 14;9(5):e95937. doi: 10.1371/journal.pone.0095937 (PMC4020745; doi:10.1371/journal.pone.0095937)
Supplement: Table S1 [file pone.0095937.s002.docx]

**Supplementary table 1. Adjusted hazard ratios (HRs) ^*^ and 95% confidence intervals (CIs) for all-cause mortality rate by summary measures^†^ of polychlorinated biphenyls (PCBs) or organochlorine (OC) pesticides, in the Prospective Investigation of the Vasculature in Uppsala Seniors (PIVUS) study**

|  |  |  | **Status of cigarette smoking** | | | **p for trend** | **p for interaction** |
| --- | --- | --- | --- | --- | --- | --- | --- |
|  |  |  | **Never smokers** | **Former smokers** | **Current smokers** |  |  |
| **Summary measure of 16 PCBs** | | |  |  |  |  |  |
| 1^st^ tertile |  | Cases/No | 14/159 | 17/138 | 7/31 |  |  |
|  |  | Adjusted HR(95%CI) | Referent | 1.2 (0.6-2.4) | 2.4 (0.9-6.0) | 0.11 | 0.05 |
| 2^nd^ tertile |  | Cases/No | 20/160 | 12/137 | 2/32 |  |  |
|  |  | Adjusted HR(95%CI) | Referent | 0.7 (0.3-1.5) | 0.4 (0.1-1.9) | 0.19 |  |
| 3^rd^ tertile | | Cases/No | 6/152 | 21/135 | 12/42 |  |  |
|  |  | Adjusted HR(95%CI) | Referent | 3.9 (1.6-9.8) | 7.1 (2.6-19.4) | <0.01 |  |
| **Summary measure of 3 OC pesticides** | | |  |  |  |  |  |
| 1^st^ tertile |  | Cases/No | 15/161 | 20/132 | 4/35 |  |  |
|  |  | Adjusted HR(95%CI) | Referent | 1.8 (0.9-3.7) | 0.9 (0.3-2.8) | 0.54 | 0.14 |
| 2^nd^ tertile |  | Cases/No | 21/174 | 12/127 | 10/28 |  |  |
|  |  | Adjusted HR(95%CI) | Referent | 0.7 (0.3-1.4) | 3.1 (1.4-6.6) | 0.10 |  |
| 3^rd^ tertile | | Cases/No | 4/136 | 18/151 | 7/42 |  |  |
|  |  | Adjusted HR(95%CI) | Referent | 4.1 (1.4-12.6) | 5.7 (1.7-19.8) | <0.01 |  |

^*^Hazard Ratios (HRs) adjusted for gender, BMI, exercise, and alcohol consumption

^†^ Values of compounds belonging to in each summary measure were individually ranked; the rank orders of the individual POPs were summed to calculate summary measures and the summaries were divided into tertiles.
